# General practitioners' perspectives on household energy insecurity across healthcare settings in Henan Province, China

**DOI:** 10.3389/fpubh.2026.1811619

**Published:** 2026-05-28

**Authors:** Xiaoyu Liu, Yang Zhang, Yufen Wei, Xiaohui Liu, Liutong Li, Cong Wang, Shan Yang, Zhuozhuo Ren, Dongyang Gao

**Affiliations:** 1 Department of General Medicine, Henan Provincial People's Hospital, Zhengzhou, Henan, China; 2 Department of Respiratory Medicine, People's Hospital of Jiaozuo City, Jiaozuo, Henan, China; 3 Department of Biostatistics, Mailman School of Public Health, Columbia University, New York, NY, United States

**Keywords:** climate change, environmental health, general practitioners, healthcare system, household energy insecurity

## Abstract

**Background:**

Household energy insecurity is a growing public health concern driven by rising utility costs, poor housing, and climate change, yet remains poorly understood in China. As frontline providers, general practitioners (GPs) are well-positioned to offer insights into community-level energy hardship across healthcare settings.

**Methods:**

A 2024 cross-sectional survey of 1,303 GPs in Henan Province, China, included providers from village clinics, township health centers, and county-, city-, and provincial-level hospitals. The survey assessed whether GPs had encountered patients reporting difficulty paying energy bills, utility shut-offs, home too hot/too cold, leaving their home due to unhealthy temperatures, or use of polluting fuels.

**Results:**

Overall, 31% of GPs encountered patients struggling to pay utility bills, and 24% reported utility shut-offs. Village-level GPs had the highest prevalence (43% and 34%), while city- and provincial-level GPs had the lowest. Coal use for warmth varied by institution (*p* = 0.002). Compared with provincial-level GPs, township- and village-level GPs had higher odds of encountering patients with difficulty paying utility bills (aOR = 1.53, 95% CI: 1.02–2.29; aOR = 2.08, 95% CI: 1.27–3.43) and utility shut-offs (aOR = 2.10, 95% CI: 1.33–3.36; aOR = 2.47, 95% CI: 1.42–4.32); village-level GPs had non-significant odds for additional warmth (aOR = 1.23, 95% CI: 0.73–2.08).

**Conclusions:**

Household energy insecurity remains a major concern in China, especially in rural areas. Village GPs are uniquely positioned to identify patients facing energy hardship. Integrating screening into primary care and improving access to clean, affordable energy are keys to advancing health equity and energy justice.

## 1 Introduction

Household energy insecurity is defined as the inability to access adequate, affordable, and reliable energy to meet basic household needs (1, 2). It is driven by rising utility costs, inadequate housing conditions, and the compounding effects of climate change, such as heat waves, cold snaps, and winter storms, which are putting households under growing pressure to maintain safe indoor temperatures (3). Sustained energy insecurity affects not only physical comfort and economic stability but also health outcomes, particularly through pathways involving indoor air quality, nutrition, mental health, and the capacity to maintain safe housing environments (4–10). However, little is known about the burden, manifestations, and health implications of household energy insecurity in China.

China is the largest developing country in the world, and its rapid urbanization and economic development have led to substantial improvements in infrastructure and energy access (11), achieving 100% electrification in 2015 (12). However, households are still affected by energy poverty, and significant disparities persist across regions, urban-rural settings, and socioeconomic groups (13–15). Rural households and low-income urban residents may still experience energy shortages, high utility bill costs, or dependence on polluting fuels (16). Furthermore, rising energy prices, extreme weather events, and uneven heating and cooling infrastructure create new vulnerabilities, particularly for populations with chronic health conditions, children, and the older adults (17).

In 2009, China launched a major health care reform that aimed at universal coverage of essential medical care and essential public health services through community health services, in which general practitioners (GPs) played a leading role (18). Within China's hierarchical, three-tier healthcare system, GPs serve as the first point of contact for patients across multiple levels of the healthcare system, from village clinics and township health centers to county-, city-, and provincial-level hospitals (19). GPs working in these settings differ from those in primary care, as they not only provide outpatient services but also engage in teaching, research, and preventive care, and may see patients from a wide geographic area through both referral and self-referral pathways (20). Through this frontline role, GPs frequently encounter patients suffering from respiratory illnesses, cardiovascular conditions, and other health issues that may be exacerbated by household energy insecurity or inadequate housing. Given their unique positioning and frequent engagement with vulnerable populations, GPs would provide a valuable perspective on how energy insecurity manifests across different communities. Therefore, this study examines household energy insecurity through the lens of GPs working at different levels of the healthcare system. This approach provides new insights into the lived realities of Chinese households, highlights implications for health and healthcare delivery, and can guide strategies to reduce energy-related disparities within China's evolving health and social policy landscape.

## 2 Methods

### 2.1 Study design and study population

This cross-sectional study was conducted in April 2024 among 1309 GPs across 18 cities in Henan Province who were attending a transition training program organized by the Henan Provincial Health Commission. The survey questionnaire was administered online via the training program's final examination platform, and participation was voluntary; respondents completed the survey either before or after the examination. A total of 1,303 GPs (99.54%) provided informed consent, completed the questionnaire, and were included in the analysis. The study was conducted in accordance with the principles of the Declaration of Helsinki and was approved by the Ethics Committee of Henan Provincial People's Hospital (Ethics No. 2025219).

### 2.2 The household energy insecurity and health survey instrument

Household energy insecurity was defined using six indicators from the household energy insecurity and health survey, a self-designed informed by previously published literatures on energy insecurity (5, 7, 10). This questionnaire collected demographic information on participating GPs, including age, sex, highest level of education, and level of healthcare institution of employment. Healthcare institutions were categorized as village clinics, township health centers, county-level hospitals, city-level hospitals, and provincial-level hospitals.

GPs were asked, in the last 12 months, whether they had encountered situations in which patients reported that they (1) struggled to pay their electricity or gas bills due to financial difficulties; (2) experienced an electricity or gas shut off due to non-payment; (3) complained that their home were too cold in the winter; (4) complained that their home were too hot in the summer;(5) temporarily left their homes due to heating or cooling issues or related costs; or (6) used coal to heat their homes for additional warmth in the winter. All items were assessed with binary (yes/no) response options. These questions were designed to capture GPs' awareness of patients' household energy challenges as encountered during routine clinical interactions.

The survey was administered via mobile phone using a link to an online platform Weijuanxing and required approximately 3 min to complete. The original questionnaire was developed in Chinese for participants. Both Chinese and English versions are provided in Supplementary material 1.

### 2.3 Statistical analysis

Descriptive statistics were calculated to summarize the characteristics of participating GPs and their responses to questions related to energy insecurity. Age was treated as continuous variable, while categorical variables, including sex, highest education level, and healthcare institution level, and all survey responses, were summarized as counts and percentages *Chi-square* tests were used to examine differences across the different levels of healthcare institution (village clinics, township health centers, county-level hospitals, city-level hospitals, and provincial-level hospitals). The energy insecurity indicators, including struggling to pay electricity or gas bills; experiencing utility shutoff due to non-payment; reporting the home being too cold in winter or too hot in summer; temporarily leaving the home due to heating or cooling issues or related costs; and using coal for additional warmth in winter, were modeled as binary outcomes in multivariable logistic regression analyses to assess the associations with the healthcare institution level, adjusting for age, sex, and education level. Forest plots were used to present adjusted odds ratios and 95% confidence intervals. All tests were two-sided, and a *p*-value < 0.05 was considered statistically significant. Statistical analyses were performed using R (version 4.3.2, R Foundation for Statistical Computing, Vienna, Austria).

## 3 Results

A total of 1,303 GPs were included in this study, including 243 from village clinics, 447 from township health centers, 167 from county-level hospitals, 229 from city-level hospitals, and 217 from provincial-level hospitals. Table 1 summarizes participant characteristics by healthcare institution levels. The median age of GPs was 42 years (IQR: 37–47), with significant variation across institution levels (*p* < 0.001). GPs from provincial- and county-level hospitals were generally older (median ages: 45 and 44 years, respectively) than those from other levels. Fifty-four percent of GPs were women, and the sex distribution varied significantly by institution level (*p* < 0.001), with men comprising a higher proportion in village clinics (56%) and women predominating in higher-level hospitals (60% in provincial-level hospitals). Educational attainment also differed significantly (*p* < 0.001), with nearly all GPs in provincial-level (100%) and city-level (98%) hospitals holding an undergraduate degree or higher, compared with 88% in county-level hospitals, 47% in township health centers, and 14% in village clinics.

**Table 1:** Characteristics of general practitioners by healthcare institution levels in Henan Province.

Characteristics	Overall	Village clinic	Township health center	County-level hospital	City-level hospital	Provincial-level hospital	*p*
	(*N* = 1,303)	(*N* = 243)	(*N* = 447)	(*N* = 167)	(*N* = 229)	(*N* = 217)	
Age (years)	42 (37, 47)	40 (36, 44)	41 (37, 45)	44 (40, 50)	40 (33, 46)	45 (42, 50)	< 0.001
Sex							
Male	600 (46%)	136 (56%)	196 (44%)	82 (49%)	100 (44%)	86 (40%)	0.004
Female	703 (54%)	107 (44%)	251 (56%)	85 (51%)	129 (56%)	131 (60%)	
Education							
Technical school diploma	152 (12%)	101 (42%)	50 (11%)	1 (0.6%)	0 (0%)	0 (0%)	< 0.001
Associate degree	320 (25%)	109 (45%)	188 (42%)	19 (11%)	4 (1.7%)	0 (0%)	
Bachelor's degree or higher	831 (64%)	33 (14%)	209 (47%)	147 (88%)	225 (98%)	217 (100%)	

GPs' reports of patients experiencing household energy insecurity by healthcare institution types are shown in Table 2. Overall, 31% of GPs reported hearing from patients who had difficulty paying utility bills, and 24% reported that patients had experienced utility shut-offs due to non-payment. These proportions varied significantly across institution levels (*p* < 0.001). GPs from village clinics reported the highest prevalence of difficulty paying bills (43%) and utility shut-offs (34%), whereas those from city- and provincial-level hospitals reported the lowest (21%−26% and 16%, respectively). Notably, reports of patients using coal for additional warmth also differed significantly across institution levels (*p* = 0.002), with the highest prevalence reported by GPs from village clinics and provincial-level hospitals.

**Table 2:** General practitioners who reported hearing of patients experiencing household energy insecurity, by the level of healthcare institution in Henan Province.

Characteristics	Overall	Village clinic	Township health center	County-level hospital	City-level hospital	Provincial-level hospital	*p* ^*^
Difficulty paying							< 0.001
No	894 (69%)	139 (57%)	301 (67%)	113 (68%)	180 (79%)	161 (74%)	
Yes	409 (31%)	104 (43%)	146 (33%)	54 (32%)	49 (21%)	56 (26%)	
Utility shut off due to non-payment							< 0.001
No	996 (76%)	160 (66%)	327 (73%)	134 (80%)	192 (84%)	183 (84%)	
Yes	307 (24%)	83 (34%)	120 (27%)	33 (20%)	37 (16%)	34 (16%)	
Leaving home because it was too hot							0.400
No	558 (43%)	101 (42%)	195 (44%)	69 (41%)	109 (48%)	84 (39%)	
Yes	745 (57%)	142 (58%)	252 (56%)	98 (59%)	120 (52%)	133 (61%)	
Using coal for additional warmth							0.002
No	423 (32%)	63 (26%)	156 (35%)	54 (32%)	93 (41%)	57 (26%)	
Yes	880 (68%)	180 (74%)	291 (65%)	113 (68%)	136 (59%)	160 (74%)	
Home is too cold in the winter							0.094
No	473 (36%)	83 (34%)	178 (40%)	54 (32%)	91 (40%)	67 (31%)	
Yes	830 (64%)	160 (66%)	269 (60%)	113 (68%)	138 (60%)	150 (69%)	
Home is too hot in the summer							0.300
No	463 (36%)	79 (33%)	168 (38%)	55 (33%)	91 (40%)	70 (32%)	
Yes	840 (64%)	164 (67%)	279 (62%)	112 (67%)	138 (60%)	147 (68%)	

^*^Pearson's Chi-squared test.

**Figure 1:**
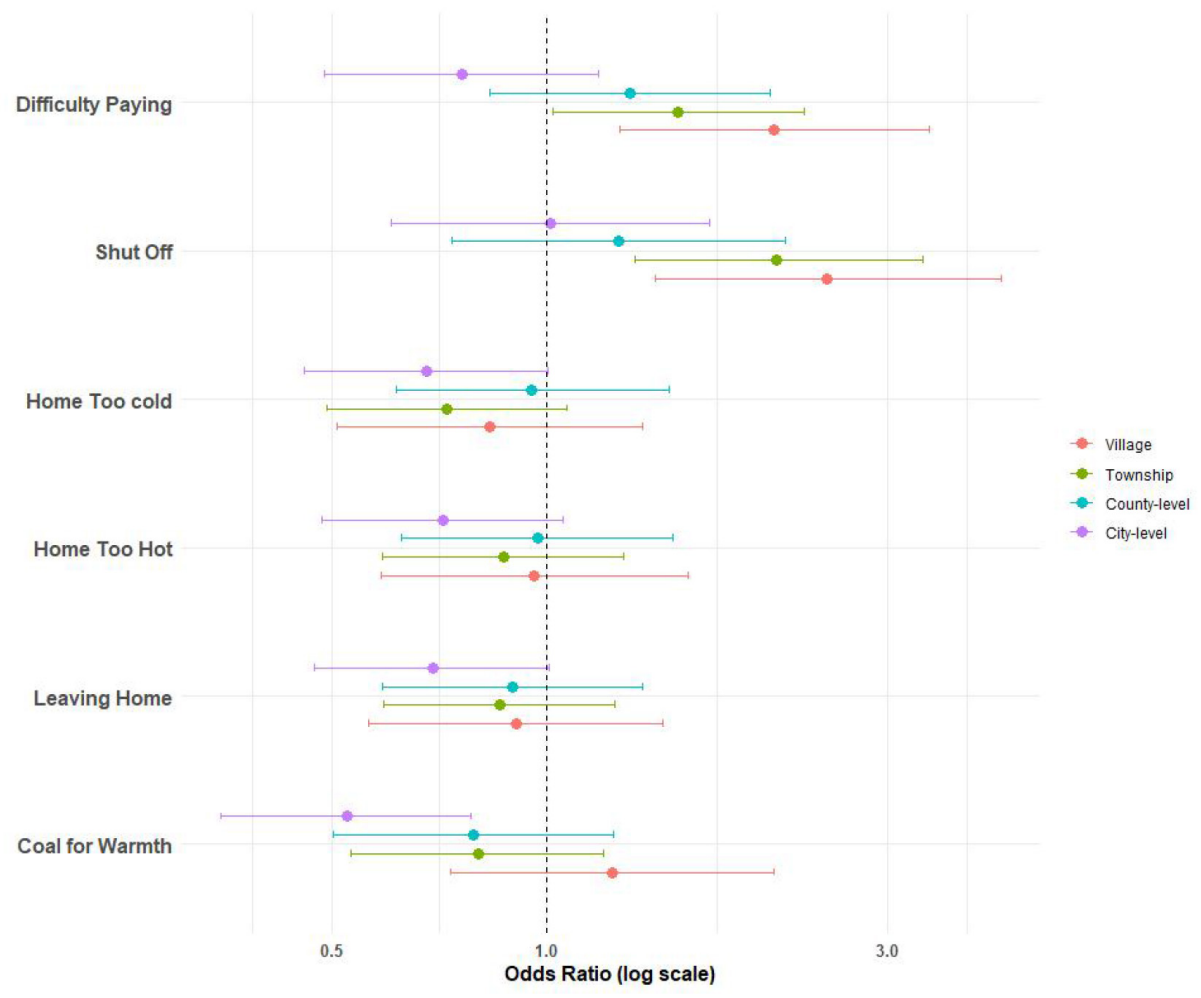
Adjusted odds ratios and 95% confidence intervals for the association between healthcare institution level and general practitioners' likelihood of encountering patients with household energy insecurity.

A multivariable logistic regression analysis was conducted to examine the associations between healthcare institution level and GPs' likelihood of encountering patients experiencing household energy insecurity. As shown in Figure 1, after adjusting for GPs' age, sex, and education level, township- and village-level GPs had significantly higher odds of encountering patients experiencing difficulty paying utility bills (township level: OR = 1.53, 95% CI: 1.02–2.29; village level: OR = 2.08, 95% CI: 1.27–3.43) and utility shut-offs (township level: OR = 2.10, 95% CI: 1.33–3.36; village level: OR = 2.47, 95% CI: 1.42–4.32) compared with provincial-level hospital GPs. Regarding the use coal for additional warmth, city-level GPs had lower odds compared to the village GPs (OR = 0.53, 95% CI: 0.35–0.78), while village-level GPs had higher odds, although this difference was not statistically significant (OR = 1.23, 95% CI: 0.73–2.08). No significant differences were observed across healthcare levels for GPs reporting patients experiencing thermal discomfort (too hot or too cold) or temporarily leaving their homes due to extreme temperatures. Detailed results for all outcomes are presented in Supplementary Table 1.

## 4 Discussion

This study provides novel evidence on household energy insecurity in China through the perspectives of GPs across multiple levels of healthcare institutions in Henan Province. To our knowledge, this is the first province-wide survey to characterize household energy insecurity in China, although the information was reported indirectly through GPs rather than directly from households. Nevertheless, as frontline healthcare providers, GPs are uniquely positioned to observe patients' lived experiences and provide valuable insights into energy-related disparities across communities.

Household energy insecurity is a global concern that affects high- and low-income settings (21, 22), and our findings in China align with patterns observed elsewhere. In our study, approximately one-third of GPs reported that patients were struggling to pay utility bills, and one-quarter reported that patients had experienced utility shut-offs due to non-payment, highlighting substantial energy-related hardship among households. Similarly, in the United States, one-third of households experienced energy insecurity in 2020, including challenges paying utility bills or maintaining adequate indoor temperatures (23). Beyond high-income countries (4), a related form of energy insecurity is widespread in developing regions, where many households lack reliable access to electricity or clean cooking fuels. Surveys in sub-Saharan Africa found that over half of households in Ghana and Nigeria experienced energy-related hardship, which was associated with stress, adverse respiratory outcomes, and barriers to healthcare access (24, 25). These findings highlight that household energy insecurity in China reflects a broader, worldwide challenge, underscoring the need for policies and interventions to promote energy equity.

Energy insecurity was more frequently reported by township and village GPs than by those working in higher-level hospitals, with the highest prevalence of difficulty paying bills and utility shut-offs observed among village clinics. In contrast, GPs from city- and provincial-level hospitals reported the lowest prevalence. This pattern aligns with studies showing that multidimensional energy poverty is significantly higher in rural areas than in urban areas in China, driven by lower incomes (26), limited non-agricultural employment, and restricted access to modern energy insecurity (27). Globally, energy insecurity disproportionately affects low-income and rural households, reflecting broader structural and economic inequalities that limit income, wealth accumulation, and housing quality (28). In China, village GPs typically serve rural populations with lower household incomes (26), less-developed energy infrastructure, and limited access to clean and affordable fuels, which may heighten vulnerability to energy-related hardship (14).

Notably, village clinic and provincial-level hospital GPs reported the highest prevalence of patients using coal for additional warmth, underscoring continued reliance on polluting fuels even among patients accessing higher-level healthcare services. This finding may reflect a combination of factors, including referral patterns, patient mobility, and potential reporting differences across healthcare settings. The composition of GP patient populations differs by healthcare level: village GPs primarily serve local residents, while provincial (tertiary) hospital GPs see patients from a broader geographic area, including both direct walk-ins and referrals from lower-level facilities (29, 30). Patients referred from rural areas may continue to rely on coal for heating, which could contribute to the unexpectedly high prevalence observed among provincial hospital GPs. This finding highlights persistent rural-urban disparities in access to clean energy sources (31) and suggests that energy insecurity among rural residents can have downstream health and service delivery implications even at tertiary healthcare institutions (13).Thermal discomfort, where homes are too cold in winter or too hot in summer, was commonly reported by GPs across all institution types, suggesting that exposure to extreme indoor temperatures is a widespread concern beyond socioeconomic status. Henan Province, located in central China, lies within the country's hot-summer/cold-winter climatic zone, characterized by extreme seasonal temperature variation that challenges indoor thermal comfort (32). This province remains economically less developed than many eastern coastal regions. Research on rural Chinese dwellings demonstrates that many homes, particularly older or poorly insulated ones, fail to maintain comfortable temperatures due to limited access to adequate heating and cooling, economic constraints, and insufficient infrastructure (33). Meanwhile, reliance on coal and other solid fuels for domestic energy continues to contribute to substantial indoor air pollution and related health burdens. These climatic, socioeconomic, and energy access conditions reinforce the need for policies that promote clean, affordable energy to mitigate health risks associated with indoor thermal discomfort and air pollution (34).

The findings also emphasize the importance of strengthening the capacity of township and village GPs, who were far less likely to have bachelor-level education, with most holding technical school or associate degrees (35). This disparity reflects structural differences in training opportunities and may influence the effectiveness of healthcare delivery, particularly in addressing social and environmental determinants of health (36). GPs in China often maintain high levels of trust and close, long-term relationships with their patients (37–39), and they are uniquely positioned to observe households' living conditions and energy-related hardships. Providing township and village GPs with targeted training and resources to screen for energy insecurity, recognize signs of thermal discomfort or utility hardship, and offering practical advice on energy-saving practices to enhance their ability to mitigate energy-related health risks.

Beyond individual-level capacity building, broader systemic strategies are also important. GPs should be involved in community-level advocacy for clean and affordable energy, and implementing policies that improve household energy security in rural areas could collectively promote energy equity. Together, these efforts have the potential to reduce health inequities and promote safer, more comfortable living environments across Henan Province.To our knowledge, this is the first study to explore household energy insecurity in China from the perspective of health workers. Previous studies in Chinese households, primarily using secondary data, have shown that multidimensional energy poverty is significantly higher in rural than in urban areas and disproportionately affects vulnerable populations, particularly middle-aged and older adults (15); however, these studies did not use primary data. Leveraging GPs' experiences provides a unique and pragmatic lens on energy-related hardship that may not be readily captured through traditional household surveys, particularly in resource-limited or hard-to-reach populations. Moreover, this province-wide scope of this survey enhances the diversity of healthcare settings and patient populations represented.

This study has several limitations. First, reliance on GPs' perceptions rather than direct household reports may introduce recall or reporting bias, and differences in doctor-patient contact frequency and context across the healthcare levels may affect comparability. Village-level GPs may maintain closer relationships with patients, which may lead to increased awareness of household energy hardships compared with GPs in higher-level urban facilities; in contrast, patients in urban areas report higher levels of trust in their GPs, which may influence reporting patterns differently across settings (37). Additionally, GPs participating in a specific training program may introduce potential bias. Future studies should incorporate direct surveys from patients to validate and complement provider-reported information and further explore urban-rural differences. Second, the cross-sectional design precludes causal inference. Longitudinal designs would help clarify causal pathways between energy insecurity and health outcomes. Third, we did not collect data on patient origin (e.g., referral vs. walk-in, or rural vs. urban residence), which limits our ability to fully explain this pattern. Future studies should collect detailed information on patient sources and care pathways to better understand differences across healthcare levels and to validate these interpretations. Fourth, the survey instrument was self-designed and not formally validated or tested for reliability. Future work will include pilot testing and psychometric evaluation to ensure the validity and reliability of the questionnaire. Finally, the study was conducted in Henan Province and may not be fully generalizable to other regions of China, but the province-wide scope provides valuable insights into household energy insecurity from a healthcare provider perspective. Expanding this work to other regions and integrating qualitative approaches could further elucidate contextual factors and inform targeted interventions to reduce energy-related health disparities.

## 5 Conclusions

Household energy insecurity remains a significant concern in China, especially among rural residents. Difficulties paying utility bills and reliance on coal for warmth highlight persistent socioeconomic and infrastructural disparities. Township and village GPs are well-positioned to identify patients facing energy-related hardship and its health effects. Integrating energy insecurity screening into primary care and connecting patients with social or energy assistance programs may help mitigate risks. Strengthening policies to ensure access to clean, affordable energy and targeted support for vulnerable households is essential to promote health equity and energy justice in China.

## Data Availability

The raw data supporting the conclusions of this article will be made available by the authors, without undue reservation.
