# Supplementary material for: General practitioners' perspectives on household energy insecurity across healthcare settings in Henan Province, China
Source: Front Public Health. 2026 May 28;14:1811619. doi: 10.3389/fpubh.2026.1811619 (PMC13253614; doi:10.3389/fpubh.2026.1811619)
Supplement: Supplementary file 1 [file Supplementary_file_1.docx]

**[English Version]**

Household Energy Insecurity and Health Survey

Thank you for taking the valuable time to participate in this household energy and health survey. **Household energy insecurity refers to the inability of a household's energy supply to adequately meet basic household energy needs. The term "household energy" herein refers to natural gas, electricity, coal, etc., used for household lighting, cooling, heating, household appliances, and electronic devices.**

This web survey is part of a research study to find out if you had ever encountered situations in which patients experienced energy insecurity and energy-related coping strategies. Participation is voluntary; you may cease filling out the questionnaire at any time if you feel uncomfortable or unwilling to continue. To ensure data authenticity, we kindly request your earnest responses.

Before taking part in the survey, please read the following information and then indicate your agreement to participate by selecting “I consent” below.

**Consent to Participate**

**“I have read and understand the above information. I agree to participate in this study with the understanding that I may choose not to participate or to stop participating at any time.”**

If you consent to participate, please click “I consent” below to continue with the survey. If you choose not to participate please click “Exit” and then the “NEXT” button to exit the survey.

( ) I consent

( ) Exit

1. What is your gender?

A. Male

B. Female

1. What is your age? _______ (years)
2. What is your highest level of education?
3. High school education or equivalent
4. Technical school diploma, no degree
5. Associate degree
6. Bachelor's degree or higher
7. What is the level of the hospital or institution where you are currently employed?
8. Village clinic / Rural clinic
9. Township health center/community health center
10. County-level hospital
11. Municipal/City-level hospital
12. Provincial-level hospital
13. Have you ever encountered situations in which patients struggled to pay for electricity or gas due to financial difficulties?
14. Yes
15. No
16. Have you ever encountered situations in which patients experienced an electricity or gas shut-off due to non-payment?

A. Yes

B. No

1. Have you ever encountered situations in which patients complained that their homes were too cold in the winter?

A. Yes

B. No

1. Have you ever encountered situations in which patients complained that their homes were too hot in the summer?

A. Yes

B. No

1. Have you ever encountered situations in which patients had to leave their homes temporarily due to heating or cooling issues, or because of the cost?

A. Yes

B. No

1. Have you ever encountered situations in which patients had to use a coal stove to heat their homes for additional warmth in the winter?

A. Yes

B. No

**【中文版】**

家庭能源与健康问卷调查

感谢您抽出宝贵时间参与本次家庭能源与健康调查。家庭能源保障不足是指家庭无法充分满足其基本能源需求。这里的家庭能源包括用于照明、制冷、取暖以及家用电器和电子设备运行的电力、天然气、燃煤等。

本调查旨在了解您在与患者交流过程中，是否听到过患者关于家庭能源使用方面的困难，例如电费、燃气费负担、冬季保暖情况、住房保温条件，以及是否采取了相应的应对策略。

本问卷遵循自愿参与原则，您可根据自身意愿选择是否继续填写，并可随时退出。为保证数据的真实性和研究质量，恳请您根据实际情况认真作答。衷心感谢您的支持与配合！

参与调查前，请先阅读以下资料，然后在下方选择“我同意”以表示同意参与。

“我已经阅读并理解了上述信息。我同意参与这项研究，但有一项谅解，即我可以选择不参与或随时停止参与。”如果您同意参与，请选择下面的“我同意”继续调查。如果您选择不参与，请选择“退出”，然后退出调查。

( ) 我同意

( ) 退出

1. 您的性别是

A. 男

B. 女

1. 您的年龄 _______ (岁)
2. 您的学历是
3. 高中及以下
4. 中专或技校
5. 大专
6. 本科及以上
7. 您单位级别是
8. 村卫生室
9. 乡镇医院或社区卫生服务中心
10. 县级医院
11. 地市级医院
12. 省级医院
13. 在与患者交流过程中，您是否听到过患者提及因经济压力而担心无法支付电费或燃气费？
14. 是
15. 否
16. 在与患者交流过程中，您是否听到过患者提及其住所曾发生过断电或端燃气的情况？
17. 是
18. 否
19. 您在与患者交流时，是否听到过患者抱怨冬天家里很冷？
20. 是
21. 否
22. 您在与患者交流时，是否听到过患者抱怨夏天家里很热？
23. 是
24. 否
25. 您在与患者交流时，是否听到患者因为家里太冷或太热，而去商场或其他地方取暖/避暑？
26. 是
27. 否
28. 您在与患者交流时，是否听到患者因为家里太冷，用煤炉取暖？
29. 是
30. 否
