# Supplementary material for: General practitioners' perspectives on household energy insecurity across healthcare settings in Henan Province, China
Source: Front Public Health. 2026 May 28;14:1811619. doi: 10.3389/fpubh.2026.1811619 (PMC13253614; doi:10.3389/fpubh.2026.1811619)
Supplement: Supplementary file 2 [file Table_1.docx]

| Supplementary Table 1. Associations between the healthcare institution levels and general practitioners’ likelihood of encountering patients experiencing household energy insecurity | | | | | | | | | | | | |
| --- | --- | --- | --- | --- | --- | --- | --- | --- | --- | --- | --- | --- |
| **Level of healthcare institution** | Difficulty paying | | Utility shut off due to non-payment | | Home is too cold in the winter | | Home is too hot in the summer | | Leaving home because it was too hot or too cold | | Using coal for additional warmth | |
|  | OR (95% CI)* | *P* | OR (95% CI)* | *P* | OR (95% CI)* | *P* | OR (95% CI)* | *P* | OR (95% CI)* | *P* | OR (95% CI)* | *P* |
| Provincial-level Hospital | 1.00 |  | 1.00 |  | 1.00 |  | 1.00 |  | 1.00 |  | 1.00 |  |
| City-level Hospital | 0.76 (0.49, 1.18) | 0.226 | 1.01 (0.61, 1.69) | 0.965 | 0.68 (0.46, 1.00) | 0.053 | 0.72 (0.48, 1.06) | 0.093 | 0.69 (0.47, 1.01) | 0.057 | 0.53 (0.35, 0.78) | 0.002 |
| County-level Hospital | 1.31 (0.83, 2.06) | 0.24 | 1.26 (0.74, 2.15) | 0.397 | 0.95 (0.62, 1.48) | 0.834 | 0.97 (0.63, 1.5) | 0.893 | 0.9 (0.59, 1.36) | 0.604 | 0.79 (0.5, 1.24) | 0.307 |
| Township Health Center | 1.53 (1.02, 2.29) | 0.04 | 2.1 (1.33, 3.36) | 0.002 | 0.73 (0.49, 1.07) | 0.104 | 0.87 (0.59, 1.28) | 0.481 | 0.86 (0.59, 1.25) | 0.429 | 0.8 (0.53, 1.20) | 0.288 |
| Village Clinic | 2.08 (1.27, 3.43) | 0.004 | 2.47 (1.42, 4.32) | 0.001 | 0.83 (0.51, 1.36) | 0.464 | 0.96 (0.59, 1.57) | 0.877 | 0.91 (0.56, 1.46) | 0.684 | 1.23 (0.73, 2.08) | 0.428 |
| *: Adjusted for the general practitioners’ age, sex and education level. | | | | | | | | | | | | |
